# Introduction to the Special Issue on Climate Ethics: Uncertainty, Values and Policy

**DOI:** 10.1007/s11948-017-9967-2

**Published:** 2017-09-09

**Authors:** Sabine Roeser

**Affiliations:** Ethics and Philosophy of Technology Section, Department of Values, Technology, and Innovation, Faculty of Technology, Policy and Management, TU Delft, Jaffalaan 5, 2628 BX Delft, The Netherlands

**Keywords:** Climate ethics, Uncertainty, Values, Policy

## Abstract

Climate change is a pressing phenomenon with huge potential ethical, legal and social policy implications. Climate change gives rise to intricate moral and policy issues as it involves contested science, uncertainty and risk. In order to come to scientifically and morally justified, as well as feasible, policies, targeting climate change requires an interdisciplinary approach. This special issue will identify the main challenges that climate change poses from social, economic, methodological and ethical perspectives by focusing on the complex interrelations between uncertainty, values and policy in this context. This special issue brings together scholars from economics, social sciences and philosophy in order to address these challenges.

Climate change is a pressing phenomenon with huge potential ethical, legal and social policy implications. Climate change gives rise to intricate moral and policy issues as it involves contested science, uncertainty and risk. In order to come to scientifically and morally justified, as well as feasible, policies, targeting climate change requires an interdisciplinary approach.

This special issue will identify the main challenges that climate change poses from social, economic, methodological and ethical perspectives by focusing on the complex interrelations between uncertainty, values and policy in this context. This special issue brings together scholars from economics, social sciences and philosophy in order to address these challenges.

The first three contributions critically discuss climate policies from a normative perspective. Idil Boran discusses recent transformations of the architecture of climate agreement under the UNFCCC. Steve Vanderheiden provides for an ethical analysis of territorial carbon sinks. Behnam Taebi and Azar Safari discuss the effectiveness and legitimacy of shaming in the context of non-adherence to climate policies.

This special issue opens with an article by political philosopher Idil Boran (2017), on ‘Principles of public reason in the UNFCCC: Rethinking the Equity Framework’, in which she discusses recent international negotiations on climate policies and what they mean in terms of dealing responsibly and effectively with the uncertainties related to climate change. Between 2011 and 2015, during the Durban Platform for Enhanced Action, the focus of the UNFCCC negotiations was on a new climate agreement, in order to achieve long-term cooperation. A major change that has been discussed in that phase was a shift away from negotiating targets for developed countries toward building the global climate effort through contributions from all countries around the world on a long term basis, through the so-called Intended Nationally Determined Contributions (INDCs). This shift gave rise to new questions about equity, which is the main focus of Boran’s paper. Sensitive to these transformations, she develops a framework on equity firmly based on a political conception of justice, whose main concern is recognition of diversity. This framework on equity, Boran contends, calls for a move away from substantive considerations of burden allocation toward procedural considerations of public reason, in order to develop guiding principles specially designed for enhancing ambition on an equitable footing over the long term.

Steve Vanderheiden’s (2017) paper ‘Territorial Rights and Carbon Sinks’ nicely links to Boran’s paper, in that he also discusses ethical issues involved with the different roles of developing and industrialized nations, specifically in the context of territorial carbon sinks. He starts with the idea of “resource privilege”, according to which the governments of developing states are claimed to have national sovereignty over the natural resources that lie within their borders. This global justice idea is also applied in the context of climate change to justify a right to extract and combust fossil fuels. However, this provides for a challenge for global climate change mitigation imperatives. Furthermore, if one were to grant national sovereignty over territorial carbon sinks this could be in tension with equitable sharing of climate mitigation burdens. Vanderheiden discusses this tension and proposes a way to reconciliation. He argues for a weaker conception of territorial rights according to which states have valid entitlement claims to some but not all of the capacities of their territorial sinks.

While Vanderheiden’s paper is focused on the legal and ethical obligations of nations, Behnam Taebi and Azar Safari’s (2017) contribution ‘On the effectiveness and legitimacy of ‘shaming’ as a strategy for combatting climate change’ focuses on the role of Multinational Corporations. They argue that while states have agreed to substantial reduction of emissions in the Paris Agreement, the success of the Agreement strongly depends on the cooperation of large multinationals, which may have less clear legal obligations to comply with climate change policies. This means that other approaches are needed. The authors discuss the effectiveness and moral legitimacy of voluntary approaches based on naming and shaming of multinationals by states. They discuss the ethical challenges of shaming strategies in cases where corporations have a direct relationship with consumers, with governments and with other corporations. Taebi and Safari argue that in order to be effective, institutional arrangements are inevitable, including independent measurement, monitoring and verification mechanisms.

Vanderheiden’s as well as Taebi’s and Safari’s contributions address the complex role of economic interests in climate policies. This connects with the following two contributions, which focus on climate economics. More specifically, the papers by Servaas Storm and Conrad Heilmann discuss the normative assumptions that are at play in climate economics, and which can have far reaching policy implications.

The contribution by Servaas Storm (2017), ‘How the Invisible Hand is Supposed to Adjust the Natural Thermostat: A Guide for the Perplexed’, discusses underlying values and macro-economic implications of mainstream climate economics. Storm observes that it is rather perplexing that while mainstream climate economics acknowledges the importance of global warming, it nevertheless recommends to ‘wait and see’, in order not to harm the economy. Storm provides for an analysis of the underlying normative assumptions of this view. These are assumptions that are highly contested within economics. Storm plots the diverging assumptions on two axes of a matrix, distinguishing economic approaches by their respective faith in markets versus regulations and by their views on efficiency and equity implications of climate actions. Storm argues that rather than following the neo-classical economic ideal of self-interest, we should adopt a stance of solidarity, as proposed by alternative economic approaches such as feminist, socialist and green economics, and that we should explicitly discuss the ethical and political challenges of climate change.

Conrad Heilmann’s (2017) contribution, ‘Values in Time Discounting’, continues this theme of the value-ladenness and inherent uncertainty of climate economics and discount rates. He provides for a philosophical analysis of climate economics, focusing specifically on the discussion about time discounting. Time discounting means that future costs and benefits are assigned a decreasing weight. Heilmann argues that time discounting is essentially an ethical problem in which values play a crucial role. According to Heilmann, the debates amongst climate economists are also value-laden and ethical debates. He provides an analysis to explicate the values inherent to methodologies for time discounting. This means that economics cannot provide for a value-free approach to time discounting. Heilmann proposes that the existing methodologies can be improved by explicitly distinguishing between ethical and scientific judgments concerning time discounting and making underlying ethical arguments explicit, which will also contribute to debates about these issues.

Storm and Heilmann have both argued amongst other things that climate economists should make their normative assumptions more explicit. Climate scientists also make normative assumptions, concerning their models and frameworks but also concerning research integrity. This is a topic that is studied in a survey by Dennis Bray and Hans von Storch, in which they investigated the normative orientation of climate scientists. This paper is followed by another empirical study, by Gabrielle Wong-Parodi and Wändi Bruine de Bruin, concerning climate change perceptions of the public and the normative implications for climate communication approaches.

Dennis Bray and Hans von Storch’s (2017) article ‘The Normative Orientations of Climate Scientists’ discusses an online survey the authors have conducted amongst climate scientists. This survey was set out to assess whether climate scientists subscribe to the famous ‘Merton-norms’. These norms have been developed in 1942 by Robert K. Merton, in order to capture the norms of scientific research. According to Merton (1942), these norms are what he called “Communism”, “Universalism”, “Disinterestedness”, and “Organized skepticism”, which is usually abbreviated as CUDOS. According to Bray and Von Storch’s survey, these norms are still very important for climate scientists, but they are not fully endorsed or present in their conduct. Climate scientists tend to withhold results until publication and to assign more importance to the status of an author than to the content of a paper, scientists aim to maintain property rights, and external factors have impact on how research is defined, all of which are in direct contrast with the Merton-norms.

Gabrielle Wong-Parodi and Wändi Bruine de Bruin (2017), in their contribution, ‘Informing public perceptions about climate change: A ‘mental models’ approach’’, argue that climate experts have an ethical obligation to effectively communicate to non-experts, as society at large needs to be informed about the scientific facts in order to make complex decisions. Wong-Parodi and Bruin de Bruin present the following maxims of effective communication, namely sharing communications that are truthful, brief, relevant, clear, and tested for effectiveness. They discuss various challenges for climate communication approaches to meet these maxims. The authors then present a ‘mental models’ approach that is designed to meet these challenges. Such an approach combines normative, descriptive and prescriptive research as well as an evaluation of the effectiveness of a climate communication.

The paper by Wong-Parodi and Bruine de Bruin provides for a direct link to the next two contributions, which discuss epistemic aspects of risk and uncertainty in the context of climate change and their implications for decision-making (Roser) and responsibility (Robichaud).

Dominic Roser (2017), in his article, ‘The Irrelevance of the Risk-Uncertainty Distinction’, argues for the claim that the common risk-uncertainty distinction is practically irrelevant. According to this distinction, in contexts of risk we have probabilities, while in contexts of uncertainty we don’t. Roser argues that we almost always have subjective or epistemic probabilities for policy-making. If we demand that probabilities have sufficiently high epistemic credentials, then we do not always have probabilities. Climate policy could then be understood as a case of decision-making under uncertainty, as here we do not have probabilities. However, Roser argues that if probabilities with low epistemic status are the best available probabilities, then our decision principles should make use of them, which means that the risk-uncertainty distinction is practically irrelevant. The Precautionary Principle is often proposed for contexts of uncertainty, as in the case of climate policy. Roser argues that even if we have probabilities, we should follow precautionary intuitions.

Phil Robichaud’s (2017) paper, ‘Is ignorance about climate change culpable?’, discusses whether lack of knowledge and ignorance in the case of climate change can be culpable or blameworthy. He analyses this problem by drawing on recent developments in theories of responsibility for ignorant action and how they would judge various cases of ignorance about climate change. According to “volitionist” theories, blameworthiness for ignorant action is based in blameworthy management of one’s beliefs. According to “quality of will” theories, culpability for ignorance is determined by the “quality of will” of the agent. Robichaud argues that while both theories lead to different verdicts in some cases, they both entail that agents who engage in strategic ignorance are culpable for their ignorance, and probably also for their ignorant actions. The theories diverge for cases in which the ignorant agent lives in a society that does not contribute to having veridical beliefs about climate change. Where on a volitionist approach, this can be seen as an excuse, on the quality of will approach, this is still seen as a failure to do one’s best to acquire sound knowledge. This argument is highly relevant in a time where climate change denial has been institutionalized on the highest level in the world’s most powerful democracy.

The last contribution to this special issue continues on the theme of responsibility, but focuses specifically on engineers. Rob Lawlor and Helen Morley (2017) argue in their article ‘Climate Change and Professional Responsibility: A Declaration of Helsinki for Engineers’ that engineers should develop a Declaration of Climate Action. Given the urgent challenges posed by climate change and the unique expertise and role of engineers, Lawlor and Morley argue that engineers have a special responsibility and opportunity to take the lead in diminishing climate change. Drawing on the history of the Declaration of Helsinki on medical research ethics, the authors argue that a similar kind of document is needed in the context of engineering and climate change. The Declaration of Helsinki was developed by physicians and stated their moral duties beyond what was legally required. The authors argue that analogously, the engineering profession should develop a document that commits engineers to combatting climate change. While there are codes of ethics for the engineering profession in different countries, they are rarely very prohibitive, and they do not have a specific focus on combatting climate change.

The contributions to this special issue provide for a broad range of analyses of the conceptual, ethical and practical implications of the uncertainty that is inherent to debates about climate change. Hopefully this special issue can make a contribution to a better understanding of these issues and to concrete recommendations of criteria for morally fair and practically feasible policy measures in order to address the politically, morally and scientifically complex issue of climate change. These challenges have become ever more urgent given recent international developments in which not only values are contested but also misleading and dangerous notions such as ‘alternative facts’ have made it to the fore of political decision making. The topic of climate ethics is more pressing than ever before, and it requires united efforts of scholars from various disciplines. This special issue aims to provide a contribution to this endeavor.

## References

[CR1] Boran, I. (2017). Principles of public reason in the UNFCCC: Rethinking the equity framework. *Science and Engineering Ethics*. doi:10.1007/s11948-016-9779-9. 10.1007/s11948-016-9779-9 27116037

[CR2] Bray, D., & von Storch, H. (2017). The normative orientations of climate scientists. *Science and Engineering Ethics*. doi:10.1007/s11948-014-9605-1. 10.1007/s11948-014-9605-1 PMC5636871 25381220

[CR3] Heilmann, C. (2017). Values in time discounting. *Science and Engineering Ethics*. doi:10.1007/s11948-017-9950-y. 10.1007/s11948-017-9950-y PMC5636865 28812205

[CR4] Lawlor, R., & Morley, H. (2017). Climate change and professional responsibility: A declaration of helsinki for engineers. *Science and Engineering Ethics*. doi:10.1007/s11948-017-9884-4. 10.1007/s11948-017-9884-4 PMC5636869 28281157

[CR5] Merton, R. K. (1942). *The normative structure of science in the sociology of science: Theoretical and empirical investigations*. Chicago: University of Chicago Press.

[CR6] Robichaud, P. (2017). Is ignorance about climate change culpable? *Science and Engineering Ethics*. doi:10.1007/s11948-016-9835-5. 10.1007/s11948-016-9835-5 PMC5636867 27896611

[CR7] Roser, D. (2017). The irrelevance of the risk-uncertainty distinction. *Science and Engineering Ethics*. doi:10.1007/s11948-017-9919-x. 10.1007/s11948-017-9919-x 28597223

[CR8] Storm, S. (2017). How the invisible hand is supposed to adjust the natural thermostat: A guide for the perplexed. *Science and Engineering Ethics*. doi:10.1007/s11948-016-9780-3. 10.1007/s11948-016-9780-3 PMC5636866 27116038

[CR9] Taebi, B., & Safari, A. (2017). On the effectiveness and legitimacy of “shaming” as a strategy for combatting climate change. *Science and Engineering Ethics*. doi:10.1007/s11948-017-9909-z. 10.1007/s11948-017-9909-z PMC5636864 28401508

[CR10] Vanderheiden, S. (2017). Territorial rights and carbon sinks. *Science and Engineering Ethics*. doi:10.1007/s11948-016-9840-8. 10.1007/s11948-016-9840-8 27900659

[CR11] Wong-Parodi, G., & Bruine de Bruin, W. (2017). Informing public perceptions about climate change: A “mental models” approach’. *Science and Engineering Ethics*. doi:10.1007/s11948-016-9816-8. 10.1007/s11948-016-9816-8 27752964

